# Nanoparticle carrier co-delivery of complementary antibiofilm drugs abrogates dual species cariogenic biofilm formation *in vitro*

**DOI:** 10.1080/20002297.2021.1997230

**Published:** 2021-11-25

**Authors:** Guilherme Roncari Rocha, Kenneth R. Sims, Baixue Xiao, Marlise I. Klein, Danielle S.W. Benoit

**Affiliations:** a Department of Dental Materials and Prosthodontics, São Paulo State University, São Paulo, Brazil; b Department of Biomedical Engineering, University of Rochester, Rochester, NY, USA; c Materials Science Program, University of Rochester, Ny, USA;; d Department of Orthopaedics and Center for Musculoskeletal Research, University of Rochester, Ny, USA; e Center for Oral Biology, University of Rochester, NY, USA; f Department of Chemical Engineering, University of Rochester, Ny, USA

**Keywords:** Biofilm, treatment, dental caries, *Streptococcus mutans*, *Candida albicans*

## Abstract

**Background:**

Dental caries is a multifactorial disease caused by pathogenic biofilm. In particular, *Streptococcus mutans* synthesizes biofilm exopolysaccharides, while *Candida albicans* is associated with the development of severe carious lesions.

**Aim:**

This study aimed to prevent the formation of *S. mutans* and *C. albicans* biofilms by exploiting pH-sensitive nanoparticle carriers (NPCs) with high affinity to exopolysaccharides to increase the substantivity of multi-targeted antibiofilm drugs introduced topically *in vitro*.

**Methods:**

Dual-species biofilms were grown on saliva-coated hydroxyapatite discs with sucrose. Twice-daily, 1.5 min topical treatment regimens of unloaded and drug-loaded NPC were used. Drugs included combinations of two or three compounds with distinct, complementary antibiofilm targets: *tt*-farnesol (terpenoid; bacterial acid tolerance, fungal quorum sensing), myricetin (flavonoid; exopolysaccharides inhibitor), and 1771 (lipoteichoic acid inhibitor; bacterial adhesion and co-aggregation). Biofilms were evaluated for biomass, microbial population, and architecture.

**Results:**

NPC delivering *tt*-farnesol and 1771 with or without myricetin completely prevented biofilm formation by impeding biomass accumulation, bacterial and fungal population growth, and exopolysaccharide matrix deposition (*vs*. control unloaded NPC). Both formulations hindered acid production, maintaining the pH of spent media above the threshold for enamel demineralization. However, treatments had no effect on pre-established dual-species biofilms.

**Conclusion:**

Complementary antibiofilm drug-NPC treatments prevented biofilm formation by targeting critical virulence factors of acidogenicity and exopolysaccharides synthesis.

## Introduction

Dental caries is a significant public health concern, as it is one of the most prevalent human diseases worldwide [1–3]. It is a chronic disease characterized by localized demineralization of dental structures caused by organic acid ‘deposits’ (e.g. acidic niches) formed by dental biofilm metabolic products [4]. Cariogenic biofilms are established through the concerted effort of multiple factors, including dysregulation of homeostasis within oral microbiota, host factors, and diet [5,6]. Biofilms are defined as highly dynamic microbial communities immersed in a self-produced extracellular matrix, where the main component of cariogenic biofilms are exopolysaccharides (EPS). EPS provide the acidic niches with three-dimensional (3D) structure and protection from saliva buffering and topical treatments [5,6]. Therefore, preventing biofilm formation is paramount to preventing several oral diseases, including dental caries.

*Streptococcus mutans* is widely credited with the development of dental caries. This species is acidogenic and aciduric [7] and encodes exoenzymes glucosyltransferases (Gtfs) that synthesize EPS (e.g. glucans) when sucrose is available [8]. *S. mutans* synthesizes the majority of EPS within dental biofilms [8]. Gtfs are also components of the salivary pellicle and foment adhesion and accumulation of several microorganisms [9], including *Candida albicans* that provides an abundance of binding sites for Gtfs produced by *S. mutans* [10]. *C. albicans* is the most commonly detected fungus on human mucosal surfaces and co-adheres with *S. mutans* and also commensal species [11], assisting biofilm formation [12–14] when proper sugar resources are available in the diet [15]. *C. albicans* is acidogenic, aciduric, and produces secreted aspartyl proteases [16]. These exoenzymes can degrade collagen within the dentin. Moreover, the symbiotic interactions between *S. mutans* and *C. albicans* increase acid production and extracellular glucan formation, enabling the assembly of a dense and abundant matrix rich in EPS [10,17] under acidic conditions, further increasing cariogenicity of biofilms [16].

Standard chemical therapies target microorganisms directly as individual causative agents; however, killing microbes is not the only way to prevent dental biofilms. Biofilms are formed by polymicrobial communities, where the interactions between microorganisms can increase pathogenicity [18]. In addition, the 3D EPS structure restricts saliva buffering while protecting microorganisms from therapeutic agents by limiting their diffusion [19–21]. Thus, the consequence of matrix removal would minimize acid demineralization of dental surfaces and reduce the number of microbial cells in the biofilm. Overall, this approach would reduce the severity of or delay biofilm formation, which is an attractive approach to control the formation and accumulation of pathogenic biofilms that precede tooth decay [22].

Through substantial fundamental studies of oral biofilms, it is well established that the key for controlling dental caries is prevention [23]. Chlorhexidine is a broad-spectrum bactericidal agent that suppresses mutans streptococci levels in saliva but is less effective against biofilms [24]. However, chlorhexidine treatment is not specific to cariogenic microorganisms; it causes damage in all cells, including commensal microbiota and oral tissues, resulting in dysregulated oral health, tooth pigmentation, dysgeusia, and burning sensations, and this topical treatment is not recommended for all ages [25,26]. Fluoride is the mainstay for caries prevention, but it offers insufficient protection against caries [27]. Instead, fluoride functions in the remineralization process and only has modest effects on bacterial metabolism by reducing acid production [28]. Therefore, new anti-biofilm agents especially those that reduce extracellular matrix production to delay or prevent biofilm build-up without disrupting commensal microbiota are desperately needed. Nevertheless, a pervasive challenge in antibiofilm drug delivery is maintaining exposure for a sufficient time to be effective against cariogenic biofilms. Thus, nanotechnology based on pH-responsive drug delivery systems (or nanoparticle carriers, NPCs) have been developed to increase antibiofilm efficacy by enhancing substantivity [29]; this drug delivery system has been studied for the delivery of *tt*-farnesol [30,31] and a combination of *tt*-farnesol and myricetin [29]. Notably, NPC antibiofilm activity is improved by limiting NPC diameters to 130 nm or less to increase EPS penetration [32,33].

Among compounds promising to prevent biofilm formation, this paper explores NPCs loaded with a combination of the terpenoid *tt*-farnesol, a flavonoid myricetin, and a small molecule known as compound 1771. *tt*-farnesol targets the cytoplasmatic membrane, decreasing the acid tolerance of *S. mutans* [22,34,35]. In fact, *tt*-farnesol is produced by *C. albicans* and has been shown to control phenotype shifts, for example, maintaining fungi in the yeast form at high concentrations [36,37]. Myricetin is a highly effective inhibitor of Gtfs in solution and reduces *gtfBC* gene expression [38–40]. Compound 1771 inhibits the synthesis of lipoteichoic acids in Gram-positive bacteria, hampering co-aggregation of microorganisms in the early stages of biofilm build-up [41–43]. Therefore, these agents have different, complementary mechanisms of antibiofilm action. Thus, the combination of these agents may prove to be highly effective at reducing biofilm virulence, but rapid clearance from saliva persists as a challenge for all small-molecule compounds [35,38]. Here, *tt*-farnesol, myricetin, and compound 1771 were loaded into NPC with biofilm affinity to maintain drug persistence in the oral cavity (increasing the substantivity) and improve anti-biofilm efficacy. Specifically, NPCs were used preventatively on *S. mutans* single-species biofilm and *S. mutans* and *C. albicans* dual-species biofilm *in vitro*.

## Materials and methods

### Polymer synthesis

**Figure 1.**
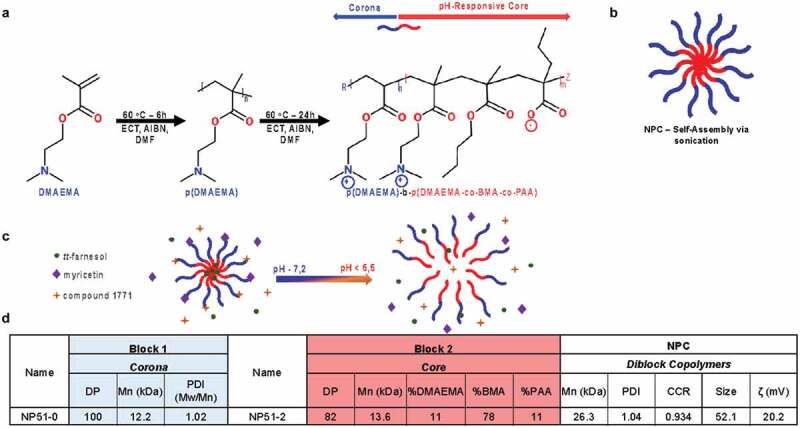
Chemical structure of diblock copolymer and the characteristics of NPC. (**A**) Scheme showing the diblock copolymer synthesis from DMAEMA to poly(DMAEMA) (Corona – blue) and a second reaction from poly(DMAEMA) to poly(DMAEMA)-b-poly(DMAEMA-co-BMA-co-PAA) (pH-responsive core – red). (**B**) Final structure after self-assembly in aqueous condition. (**C**) Representative position of drug loading within NPC and release after rapid pH drop (green sphere represents *tt*-farnesol in the core, the purple diamond represents myricetin in the corona, and the yellow star represents compound 1771 in the corona). (**D**) Characterization summary of NPC via NMR (proton nuclear magnetic resonance spectroscopy), GPC (gel permeation chromatography for molecular weights (M_w_/M_n_) and polydispersity index (PDI)), size and zeta potential (via dynamic light scattering (DLS)) results summarizing the characteristics of poly(DMAEMA) and the full diblock used in this work

Using a Reversible Addition-Fragmentation Chain Transfer polymerization (RAFT), Block 1 was prepared (Figure 1). From this block 1, a diblock copolymer was prepared (Figure 1D).

Block 1 – Cationic Corona Block Synthesis: Dimethylaminoethyl methacrylate (DMAEMA) was distilled and mixed with a specific molar ratio of 4-cyano-4-[(ethylsulfanylthiocarbonyl)-sulfanyl]pentanoic acid (ECT) and 2,2-azobisisobutyronitrile (AIBN – initiator) to start the synthesis. The desired DMAEMA:ECT:AIBN molar ratio was 450/5/1 in dimethylformamide (DMF) at 40 wt % to obtain block 1. After massing and mixing the components, the solution was placed in a reaction vessel contained within a  fume hood with a Schlenk line connected to a nitrogen tank to purge the reaction vessel for 45 min. After this period, the vessel was placed in an oil bath at 60 °C for 6 h. The next step was stopping the reaction by exposing the vessel to atmospheric oxygen. The resulting poly(DMAEMA) was collected in clean Falcon tubes and centrifuged (4,000 rpm for 10 min) with 80:20 pentane/diethyl ether (v/v) four times to wash and dry under vacuum overnight.

Diblock – Hydrophobic pH-sensible Core Block Synthesis: Block 1 [p(DMAEMA)] was mixed with AIBN as initiator and 15 wt % DMAEMA, 55 wt % butyl methacrylate (BMA), and 30 wt % propylacrylic acid (PAA) dissolved in 40 wt % DMF. After mixing the reagents, the diblock reacted in an oil bath at 60 °C for 24 h. The reaction was stopped, and the vessel was exposed to atmospheric oxygen. The resulting product was poly(dimethylmethacrylate)-b-poly(dimethylaminoethyl methacrylate-co-butyl methacrylate-co-propylacrylic acid) or p(DMAEMA)-b-p(DMAEMA-co-BMA-PAA). After multiple purifying rounds of centrifugation using 80:20 pentane/diethyl ether (v/v), the polymer went to the vacuum overnight [44–46] (scheme described in Figure 1A).

### Polymer purification and storage

The dried diblock copolymer was dissolved in 5 mL 100% ethanol and 25 mL PBS and placed in 6–8 kDa dialysis membrane tubing (Spectrum Laboratories) for 4 days with four water changes each day. The dialyzed solution was frozen at −80 °C overnight and placed in a lyophilizer (Labconco Freezone) for 4 days. After this period, the tube was warmed to room temperature prior to opening and using the nanoparticle.

### Polymer characterization

The molecular weights (M_w_/M_n_) and polydispersity’s (PDI) from block 1 and diblock were calculated using gel permeation chromatography – GPC – (Shimadzu Technologies) with a miniDOWN TREOS multi-angle light scattering detector (Wyatt Technology) in line with an Optilab T-rEX refractive index detector (Wyatt Technology). The composition and the structure of block 1 and diblock were determined using proton nuclear magnetic resonance spectroscopy – NMR – (^1^H NMR – Bruker AVANCE 400–1). Polymer size and zeta potential were measured from block 1 and diblock via dynamic light scattering (DLS) analysis in a Zetasizer Nano ZS (Malvern Panalytical). The samples were prepared at 2.7 mg mL^−1^ in PBS at pH 7.2. They were placed in a bath sonicator for 15 min. The solution was diluted in 90:10 water/nanoparticle solution (v/v), and filtered using 0,45 µm polyvinylidene difluoride (PVDF) aqueous syringe filter into a p1070 capillary cell cuvette (Malvern Panalytical) [44,46] (Figure 1D).

### Antibiofilm compounds tested

The test agents were *tt*-farnesol (*trans,trans*-3,7,11-Trimethyl-2,6,10-dodecatrien-1-ol – Sigma-Aldrich – CAS number: 106–28-5 – purity > 96%), myricetin (3,3′,4′,5,5′,7-Hexahydroxyflavone – TCI America – CAS number: 529–44-2 and product number: M2131 – purity > 97%), and compound 1771 ([(5-phenyl-1,3,4-oxadiazol-2-yl)carbamoyl]methyl 2-{naphtha [2,1-b] furan-1-yl}acetate – Chemscene – CAS number: 877,950–01-1 – purity > 98%). This study evaluated the activity from these drugs loaded into nanoparticle carriers [30,31,46]. The concentration was maintained the same in all microbiological tests being 4.5 mM *tt-*farnesol – or 1,000 µg/mL, 1 mM myricetin – or 318.24 µg/mL, 0.39 mM – or 166.67 µg/mL compound 1771, and 0.10 mM – or 2.7 mg/mL nanoparticle (NP52-1 – chosen for all tests and called NPC in the text – Figure 1D) when it was present.

**Figure 2.**
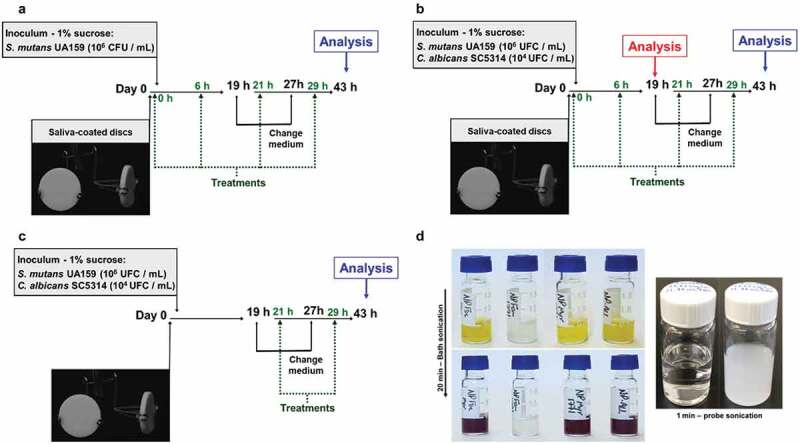
Experimental design. (**A**) Single-species biofilm treated by prevention protocol. (**B**) Dual-species biofilm treated by prevention protocol. (**C**) Dual-species biofilm treated by control protocol. (**D**) Treatments before and after bath sonication – characteristic color changes from yellow to dark purple after electrostatic interaction between NPC and myricetin, and before and after probe sonication of *tt*-farnesol in PBS to prepare the emulsion for adding the NPC in treatments that contain NPC-far-X (when X can be: no treatment or myricetin or compound 1771 or myricetin-compound1771). Green dashed arrows are the time for treatments, and solid black arrows are the time for medium changes

The characterization for *tt-*farnesol and myricetin with this polymer was published previously [29–31]. This study focuses on the characterization of the nanoparticle with the compound 1771 to verify the advantage of delivering it alone or combined with the other two drugs against *S. mutans* single-species biofilm and *S. mutans* and *C. albicans* dual-species biofilm. *tt-*farnesol was prepared in a PBS suspension (emulsion) using a probe sonicate for 30 s at 4.5 mM (or 1 mg/mL) (always prepared fresh – sonication time may vary by volume – 30 s was for 500 µL – Figure 2D). The myricetin stock solution was made using 100% DMSO at 100 mM (or 31.824 mg/mL). The compound 1771 stock solution was prepared using 20% DMSO and 80% EtOH (ethanol) at 4.68 mM (or 2 mg/mL). All treatments were stored at −20°C [29,41].

The sequence to prepare the solutions was: 1) preparing *tt*-farnesol emulsion; 2) adding the emulsion to a pre-weighed NPC; 3) adding the compound 1771; and 4) adding the myricetin in the end.

### Absorbance spectroscopy

**Figure 3.**
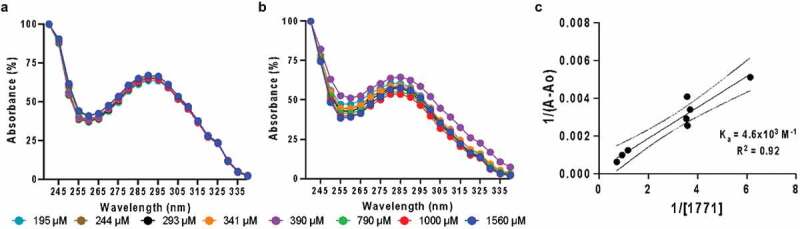
Normalized absorbance spectroscopy data revealed an interaction between NPC and compound 1771. (**A**) Representative absorbance spectra for multiple drug concentrations (1,560 µM – 195 µM) with a constant NPC concentration (195 µM – 1,560 µM) in pH 7.2 phosphate buffer. (**B**) Representative absorbance spectra for multiple free drug concentration (195 µM – 1,560 µM) in pH 7.2 phosphate buffer. (**C**) Double-reciprocal plot of absorbance data (λ = 250 nm) showing a correlation (R^2^ = 0.92) between inverse absorbance (When ‘A’ is the compound associated with NPC absorbance and ‘Ao’ is free drug absorbance) and inverse compound 1771 concentration, resulting in an association constant (y-intercept/slope, Ka) of 4.6 × 10^3^ M^−1^

Seven different concentrations of compound 1771 and compound 1771 associated with NPC (fixed concentration at 0.10 mM) were prepared for this test diluting in phosphate buffer at pH 7.2 (PBS). Using a Take3 plate and 2 µL from each sample, the Biotek Citation 5 imaging reader read the samples from 240 to 400 nm in increments of 5 nm (Figure 3). After collecting all results from compound 1771 alone (A_0_) and associated with nanoparticle (A), a graphic correlation was made using 1/(A – A_0_) vs. 1/(molar compound 1771 concentration) [47].

### Fluorescence spectroscopy

Seven different concentrations of compound 1771 associated with NPC (fixed at 0.10 mM) were prepared for this test diluting on PBS. Using a 96 black-well plate and the Bioteck Citation 5 imaging reader (Xenon Flash Light on High), the samples were read at an excitation of 280 nm from an emission wavelength of 300–620 nm, with a manual gain of 50.

### Size and zeta-potential of drug loaded NPC

Polymer size and zeta potential were measured via dynamic light scattering (DLS) analysis in a Zetasizer Nano ZS (Malvern Panalytical). The samples were prepared in phosphate buffer (PBS – pH 7.2) using different concentrations of compound 1771 associated with constant NPC concentration: 0.10 mM. Complementary samples were prepared using NPC associated with *tt*-farnesol (NPC-far), *tt*-farnesol-compound 1771 (NPC-far-1771), and *tt*-farnesol-myricetin-compound 1771 (NPC-far-myr-1771). The samples were diluted 90:10 in water/nanoparticle solution and filtered using an 0.45 µm PVDF aqueous syringe filter into p1070 capillary cell cuvettes (Malvern Panalytical) [44,48].

### Characterization of NPC drug loading

The characterizations of NPC loaded with *tt*-farnesol and myricetin were described previously [29–31,46,48]. For loading the compound 1771, a solution was prepared with pre-weighed polymer (NPC) dissolved on PBS at 2.7 mg/mL, and placed in a bath sonication for 20 min. The compound 1771 was added at the final concentration of 390 µM. The solution was sonicated using a probe at 7–10 watts for 30 s. Free compound 1771 were purified, and NPCs loaded were concentrated using 10 kDa centrifugal filters [Pall Omega (modified polyethersulfone) membrane – product ID = MAP010C36 – pore size 10 kDa]. The concentrate was recovered to initial volume adding PBS to complete 0.5 mL, and an equal amount of methanol was added and filtered using an 0.45 μm PVDF aqueous syringe filter before running it in the the HPLC to measure the concentration of compound 1771 loaded in the NPCs. The HPLC test was set up to 20 μL injection volume, and it was conducted using a mobile gradient phase consisting of HPLC-grade 90% methanol and 10% water and a Kromasil C18 column (50 mm x 4.6 mm, 5 μm particle size, 100 Å pore size from Supelco, Bellefonte, PA) with a flow rate of 0.5 mL/min over 20 min. The column effluent was monitored with a variable wavelength UV−vis detector at 210 nm and 250 nm (Shimadzu Technologies). The relative area-under-the-curve values for the compound 1771 peaks that occur between ∼6 and 7 min were compared with a standard curve to determine the loaded concentration. The drug loading efficiency was calculated after comparing the difference between the initial concentration added in the solution and the results from loaded concentration [DLE = 100% x (wt_loaded_/wt_0_), where wt_loaded_ is the amount of the loaded drug and wt_0_ is the initial amount of the drug used].

### Characterization of the NPC drug release

The characterization of the NPC drug release was made using a standard dialysis protocol [30,31,48]. After drug loading (as described above), 1 mL from this solution was collected and stored at −80 °C (t_0_); the remaining solution was placed into two different prewetted 6 − 8 kDa dialysis membrane tubings (Spectrum Laboratories). The first membrane was placed in a 300 mL beaker with phosphate buffer at pH 4.5 and the second membrane in pH 7.2, both at 37 °C. Six more samples were collected at different times: 2, 4, 8, 12, 24, and 48 h. All samples were immediately frozen and stored at −80 °C until the drug concentration of each sample was assessed via HPLC as described above for drug-loading characterization, comparing the results with the standard curve. Release rate constants (k) and release half-times (t_1/2_) were calculated according to equation [% release = 100 x (1 − e^−k.t^)], where % release is the percent of drug release at time (t) and k is the observed kinetic constant of drug release (k_obs_) following to t_1/2_ = ln(2)/k_obs_.

### Antimicrobial activity tests

The antimicrobial activity tests were performed with the planktonic cultures of *S. mutans* UA159 (ATCC 700610) to define the minimum inhibitory and bactericidal concentrations (MIC and MBC, respectively) and *C. albicans* SC5314 (ATCC MYA-2876) to determine the minimum inhibitory and fungicidal concentrations (MIC and MFC, respectively). The concentrations of the drugs were tested in duplicates, and the experiments for each microorganism were repeated three times. Thus, this study was focused on whether or not to use nanotechnology against each strain. The initial concentration of microorganisms was 10^6^ CFU/mL and they were grown in 96-well flat-bottom plates using 200 µL per well for 24 h at 37°C in 5% CO_2_ (Wiegand et al., 2018). The treatments tested were 1) NPC associated with *tt-*farnesol, myricetin, and compound 1771; 2) NPC associated with *tt-*farnesol and myricetin; 3) *tt-*farnesol, myricetin, and compound 1771 free; 4) NPC. Every well was diluted in 10-fold NaCl 0.89% and plated on blood agar plates. The plates were incubated (48 h at 37 °C in 5% CO_2_), and the colonies were counted.

### Saliva collection, preparation, and salivary pellicle formation

The stimulated whole human saliva was collected by chewing a piece of parafilm. The saliva was maintained on ice during collection. The donor rinsed the mouth with deionized water before starting the collection. The first 5 mL collected were discarded. The saliva was diluted 1:1 in adsorption buffer (50 mM KCl, 1 mM KPO_4_, 1 mM CaCl_2_, 1 mM MgCl_2_, in dd-H_2_O, pH 6.5) and 1:1,000 of 0.1 M phenylmethylsulfonyl fluoride (Sigma). Next, saliva was centrifuged (4,000 rpm, 4 °C, 15 min). The clarified portion was filtered (0.22 µm low protein-binding polyethersulfone membrane filter), and aliquots were stored in a freezer at −80 °C. For salivary pellicle formation, the hydroxyapatite (HA) discs (surface area of 2.7 ± 0.2 cm^2^, Clarkson Chromatography Products Inc., PA) were placed vertically into custom-made holders (two discs per holder – Figure 2) and sterilized by autoclaving to keep the discs in an upright position during the experiment. Next, the discs were hydrated for 30 min with sterilized deionized water and transferred to 24-well plates containing filtered sterilized saliva and incubated (37 °C, 75 rpm, 1 h). Each apparatus with discs was removed from saliva, dip-washed three times into adsorption buffer, being ready as saliva-coated hydroxyapatite (sHA). The sHA were either treated or placed into a microbial inoculum, depending on the treatment regimen described in the following items [49,50].

### Experimental design

Two treatment regimens were employed: Prevention and Control.

In the prevention regimen, the sHA discs were topically treated before contact with the microbial inoculum (treatment at 0 h). For this regimen, the first step was testing the approach against a single-species biofilm using the bacterium. The second step was testing it on a dual-species biofilm model (associated with more severe cavities than the bacterium single-species model – [51]). The treatments were applied on each surface at 0, 6, 21, and 29 h (Total: four treatments – total 6 min of exposure), and the culture medium was replaced at 19 and 27 h (Figure 2A). In the control regimen, the sHA discs were placed in the dual-species inoculum, incubated, and the first topical treatment was performed after 21 h (Total: two treatments – total 3 min of exposure) (Figure 2C). The culture medium was replaced at the same period, and the pH of the spent medium was measured in both regimens. The exposure time was the same, 1.5 min, and the discs were washed by dipping them three times in 0.89% NaCl solution before and after each treatment. The first wash was to remove the excess culture media, the discs were treated, and the second wash was to remove excess treatments as a mouthwash process (to ensure that the excess did not interact with the culture medium). The volume of treatment was 200 µL being a drop of 50 µL for each surface every 45 s for each disc. Every treatment was performed in duplicate in at least three different experiments [30,38,51].

### Microbiological test

The strains *S. mutans* UA159 and *C. albicans* SC5314 were used to prepare the inoculum. The strains were grown on blood agar plates (48 h/37°C/5% CO_2_). Five to ten colonies of each microorganism were inoculated into 10 mL of culture medium: 2.5% tryptone with 1.5% yeast extract containing 1% of glucose (TYE + 1% glucose) and incubated (37°C/5% CO_2_). After 16–18 h, 1:20 dilutions of each starter culture were performed in TYE + 1% glucose medium, and the cultures were grown until mid-log growth phase (OD 600 nm 0.74 ± 0.25 for *S. mutans*, and 0.70 ± 0.05 for *C. albicans*). A dual-species inoculum was prepared with a defined population (*S. mutans* 10^6^ CFU/mL and *C. albicans* 10^4^ CFU/mL; CFU: colony forming units) in TYE with 1% sucrose (pH 7.1) and grown for 43 h (exception for confocal microscopy at 19 h). A fresh culture medium was replaced two times (19 h and 27 h), and the pH from the spent culture medium was assessed. Topical treatments were divided into two regimens as described above.

### Biofilm analyses

After 43 h, biofilms were dip-washed in saline solution (0.89% NaCl). Each biofilm (disc) was transferred to a glass tube containing 2 mL of saline solution. The glass tubes with biofilms/discs were placed inside a Becker containing distilled water and subjected to water bath sonication for 10 min. A sterilized metal spatula was used to scrape off any remaining biofilm from each disc surface, and each biofilm suspension was collected into a 15 mL tube. Each glass tube was washed with 3 mL of saline solution, which were transferred to the tube containing the initial 2 mL, yielding a 5 mL total biofilm suspension per disc. The final suspension was sonicated using a probe at 7–10 watts for 30 s. An aliquot of 100 µL was used for a 10-fold serial dilution to determine the number of CFU/mL by plating onto blood agar plates (37 °C/5% CO_2_/48 h). The remaining suspension was centrifuged (4,000 rpm/20 min/4 °C), and each pellet was washed twice with MiliQ water. After the washes, the pellets were suspended in 2.5 mL of MilliQ water, and it was used to analyze insoluble dry-weight (biomass) [50,51].

### Confocal microscopy

For confocal microscopy, the biofilms were grown on the sHA discs and treated following the prevention regimen described above. Also, 1 µM dextran conjugated with Alexa Fluor 647 (ThermoFisher – D22914) was added to the culture medium since the beginning of the experiment to label the exopolysaccharides in the matrix (red color) [52]. After 19 and 43 h, the biofilms were rinsed using sterilized 0.89% NaCl, transferred to wells containing 2.6 µM Syto9 (ThermoFisher – S34854) in 0.89% NaCl for 30 min to label the nucleic acid of bacterial and fungal cells (green color). The samples were placed in the NaCl 0.89% solution until imaging using Andor Dragonfly Spinning Disc Confocal and the Fusion software to acquire the images. The ImarisViewer software was used to process the final images.

### Scanning electroscope microscopy

The experiment and treatments followed the prevention regimen. After 43 h, the biofilm samples were placed in EM fixative (0.1 M sodium cacodylate buffered 2.5% glutaraldehyde/4% paraformaldehyde) and left submerged for 24 h at 4 °C before acquiring the images on 25, 100, 500, 1,000, 10,000, 20,000x at Zeiss Auriga field emission – SEM and the Gatan digital camera system.

### Statistical analyses

The statistical analyses were performed using Prism9 GraphPad software (GraphPad Software, Inc., La Jolla, CA), employing a significance level fixed at 5%. The data were analyzed by the Shapiro–Wilk normality test. The data with normal distribution were subjected to parametric tests ANOVA one-way, followed by Tukey’s multiple comparison test.

## Results

Using reversible addition-fragmentation chain transfer polymerization (RAFT), a diblock copolymer, poly(dimethylaminoethyl methacrylate)-b-poly(dimethylaminoethyl methacrylate-co-butylmethacrylate-co-propylacrylic acid) or p(DMAEMA)-b-p(DMAEMA-co-BMA-co-PAA), was prepared for all studies reported here (Figure 1A). Through sonication, the diblock self-assembled into micelles (Figure 1B). During self-assembly, drug loading occurred via thermodynamically favorable interactions with the various chemical moieties of the NPC (e.g. hydrophobic and electrostatic interactions). Furthermore, the pH-responsive core block imbibed control over drug release based on the microenvironment: slow release at neutral pH and rapid release at acidic pH (Figure 1C). The specific polymer composition was chosen due to its high percentage of butyl methacrylate (BMA) within the core (78%) (represented in red in Figure 1A), polydispersity index (PDI – M_w_/M_n_) and corona-to-core molecular weight ratio (CCR) close to 1 (1.04 and 0.93, respectively), and high surface charge (+20.2 mV) (Figure 1D), which we have previously established to have excellent antibiofilm delivery capabilities [29–31,48].

### Electrostatics drive compound 1771 loading within NPC

Absorbance spectroscopy was performed to analyze drug loading within NPC. Previous work showed *tt*-farnesol and myricetin loading within NPC cores and coronas, respectively [30,48]. Therefore, only compound 1771 was reported here. Data revealed that electrostatic interactions occurred between compound 1771 and the NPC corona. In particular, changes in the absorbance curve were observed using multiple concentrations of compound 1771 spanning 195–1,560 µM at a constant NPC concentration of 0.10 mM or 2.7 mg/mL. Generally, absorbances were lower for free drug in solution compared to drug-loaded NPCs (Figure 3B) with a peak shift from 285 to 290 nm for free drug versus drug-loaded NPC (Figure 3A). A shoulder also occurred between 240 and 250 nm with drug-loaded NPC that was absent in drug-only controls. Increases in intensities, shifts in maximum absorbance wavelengths, and appearance of shoulders indicated strong electrostatic interactions between drug and NPC. Subsequently, absorbance data were used for a double-reciprocal plot to extract an association constant (K_a_) of 4.6 × 10^3^ M^−1^. The linear correlation of K_a_ (R^2^ = 0.92) highlighted the electrostatic interaction based on a host-guest binding model between the NPC and 1771, which was concentration-dependent (Figure 3C) [47,53].

**Figure 4.**
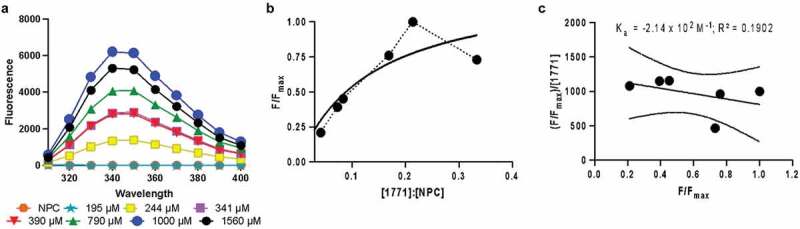
Fluorescence spectroscopy substantiated that electrostatic interactions occur between compound 1771 and NPC Corona. (**A**) Representative fluorescence spectra showing fluorescence enhancement at λ_Em_ = 340–350 nm (using λ_Ex_ = 280 nm) as the compound 1771 concentration is increased for a single NPC concentration (0.10 mM). The sample saturates above 1,000 µM. (**B**) Equilibrium binding of NPC and 1771 is measured by the increased fluorescence (λ_Ex_ = 280 nm, λ_Em_ = 620 nm). The plot shows F/F_max_ (y-axis) vs. the ratio of compound 1771 concentration to NPC concentration (x-axis) for NPCs, and the graphic highlighted the saturation point in the highest concentration (blue arrow). (**C**) Representative Scatchard plot for NPC loaded with compound 1771 in a pH 7.2 phosphate buffer, indicating an association constant (negative slope, K_a_) of ∼2.14 × 10^2^ M^−1^

Fluorescence spectroscopy was performed to confirm and give additional insight into the interaction between NPC and 1771. A single peak is indicative of one drug tautomer in solution. Based on this premise, after loading compound 1771, only one structure was observed. The reaction was concentration-dependent and saturated at the highest concentration tested (1,560 µM) with peak maxima between 340 and 345 nm (Figure 4A). The concentration used in the microbiological tests was 390 µM (or 166.7 µg/mL), which was far lower than the maximum loading concentration found here. These data were used to determine binding constants and interactions using Scatchard plots. The interaction of NPC and 1771 was k = ∼2.14 x 10^2^ M^−1^ (R^2^ = 0.1902), suggesting an electrostatic binding of drug within the NPC corona (Figure 4C).

#### NPC size increased but surface charge was maintained with drug loading

**Figure 5.**
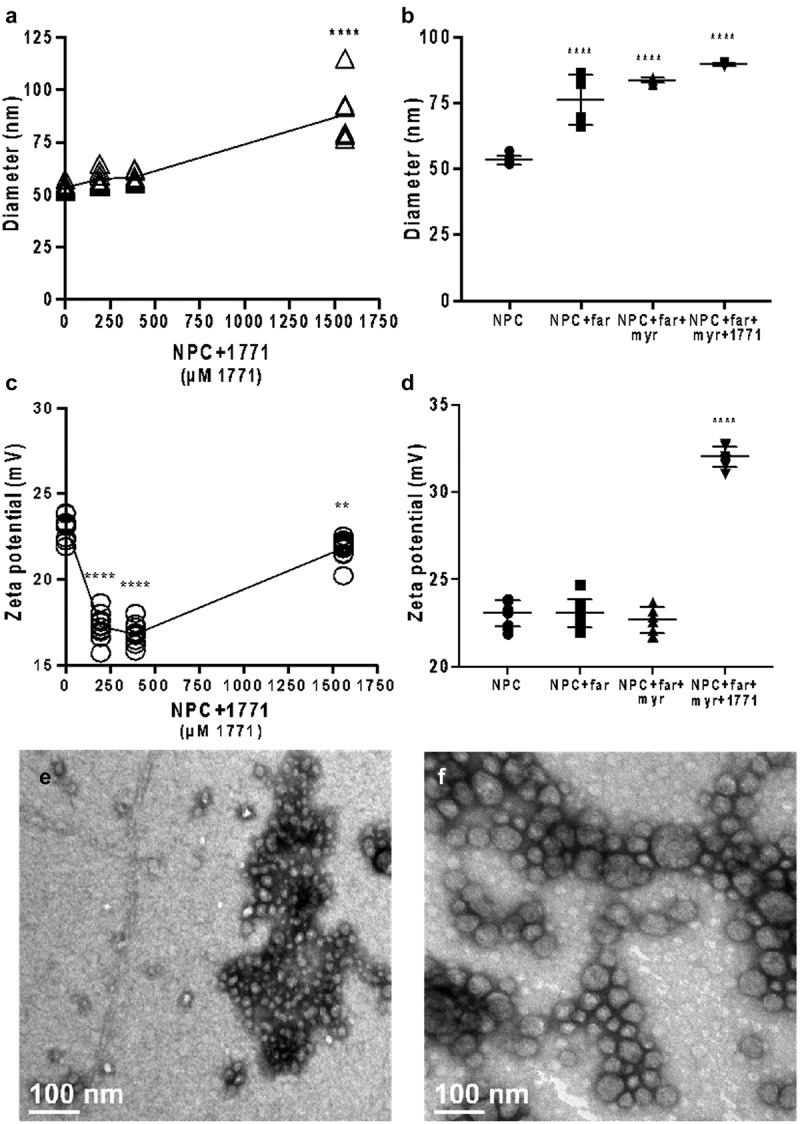
Size, zeta potential, and transmission electron microscopy (TEM) analyses confirm the interaction between compound 1771 and the NPC Corona. (**A**) Size data from NPC-compound 1771 at different concentrations; (**B**) Size data from NPC associated as different combinations of *tt*-farnesol, myricetin, and compound 1771; (**C**) Zeta potential changes after NPC loading the compound 1771 in different concentrations; (**D**) Zeta potential changes after NPC associated as different combinations of *tt*-farnesol, myricetin, and compound 1771 (concentrations used on B and D are: Far: 4.5 mM; Myr: 1 mM; 1771: 0.39 mM; NPC: 0.10 mM); (**E**) Transmission electron microscopy from NPC alone; (**F**) Transmission electron microscopy from NPC-far-myr-1771. Data are shown as mean ± standard deviation from n = 10 independent measurements. ****p < 0.0001 and **p < 0.05 compared to the NPC group. Low concentrations of compound 1771 yielded zeta potential decrease compared with NPC alone, and a high concentration of compound 1771 decreases the values less compared with low concentrations. In the presence of *tt*-farnesol, compound 1771 does not interfere in this data, and after myricetin is added, the surface charge increases to ~32 ± 0.53 mV

Size and surface charge, which impact binding to and transport within biofilms, are important parameters to characterize both unloaded and drug-loaded NPC. Neither compound 1771 nor myricetin affected the NPC diameter at concentrations used in antibiofilm tests (390 µM or 166.7 µg/mL for 1771 and 1 mM or 318.2 µg/mL for myricetin) compared to the NPC alone (concentration = 0.10 mM or 2.7 mg/mL) (Figure 5B). NPC size only changed at the highest concentration of compound 1771 (1,560 µM), increasing from 53.4 ± 1.4 nm on unloaded NPC to ∼ 89 ± 0.49 nm. *tt-*farnesol (4.5 mM or 1 mg/mL) also increased NPC size from 53.4 ± 1.4 nm to 76 ± 8.6 nm (Figure 5A), similar to our previous findings [29–31].

The effects of drug loading on the zeta potential were explored for each drug combination (Figure 5D). The NPC zeta potential showed a high positive charge on the surface (+23.08 ± 0.67 mV), which is desirable for binding anionic oral surfaces including salivary pellicle, exopolysaccharide matrix, and microbes. The addition of compound 1771 decreased the surface charge to +17.23 ± 0.75 mV. While statistically significant, previous data suggested that the observed reduction in surface charge will not alter the ability of NPC to bind to relevant surfaces [30,54] (Figure 5C). Interestingly, the zeta potential increased to +32.06 ± 0.53 mV after NPC loading *tt-*farnesol, myricetin, and compound 1771. Thus, all formulations exhibited cationic surface charges (Figure 5C-D). The transmission electron microscopy (TEM) images showing NPC alone (Figure 5E) and NPC-far-myr-1771 (Figure 5F) highlight increased NPC size after drug loading, confirming DLS observations.

*NPC exhibited high loading efficiency and capacity and modest pH-responsive release of compound 1771*

**Figure 6.**
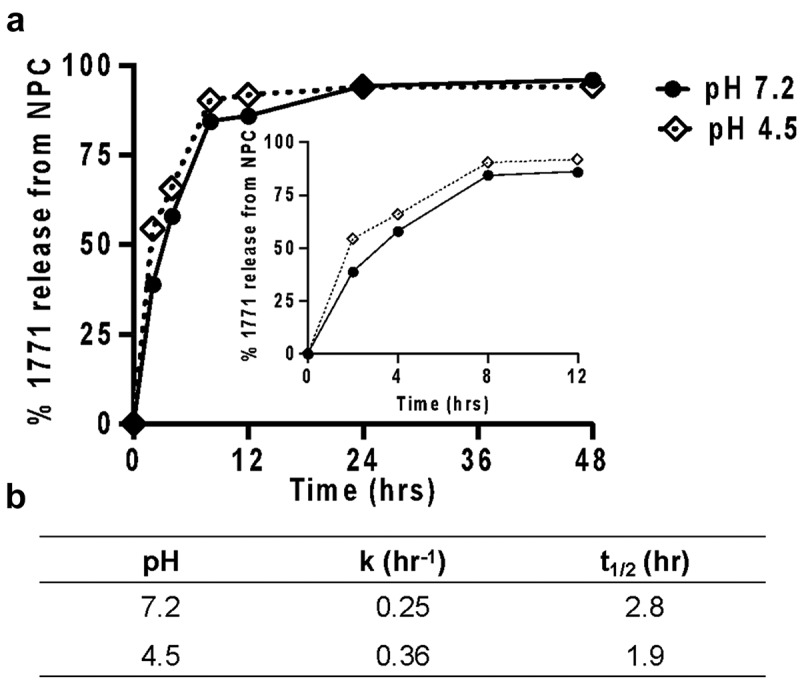
Compound 1771 loading and release from NPC. (**A**) Compound 1771 release from NPC-1771 loaded system at pH 7.2 (solid line) and pH 4.5 (square dot line) and highlights the data from early h (0 to 12). (**B**) Half-time of release (t_1/2_) and release rate constants (k) for NPC-compound 1771 from data shown in (**A**)

The drug loading efficiency (DLE) and release of 1771 were characterized. DLE represents the amount of drug that loads within NPC compared with the maximum available. For NPC-1771, DLE was found to be 96%, as measured via high-performance liquid chromatography (HPLC) (see Supporting information, Figure S1). Drug release behavior was calculated under two pH conditions; at neutral pH (7.2), ∼38% release occurred in the first h compared to ∼52% at pH 4.5. After 12 h, approximately 90% of compound 1771 was released for both pH conditions (Figure 6A). At neutral pH, the release rate constant was k = 0.25 h^−1^ (t_1/2_ = 2.8 h); at low pH, k = 0.36 h^−1^ (t_1/2_ = 1.9 h) (Figure 6B).

#### NPC-mediated drug delivery significantly improved antibacterial activity against S. mutans

**Figure 7.**
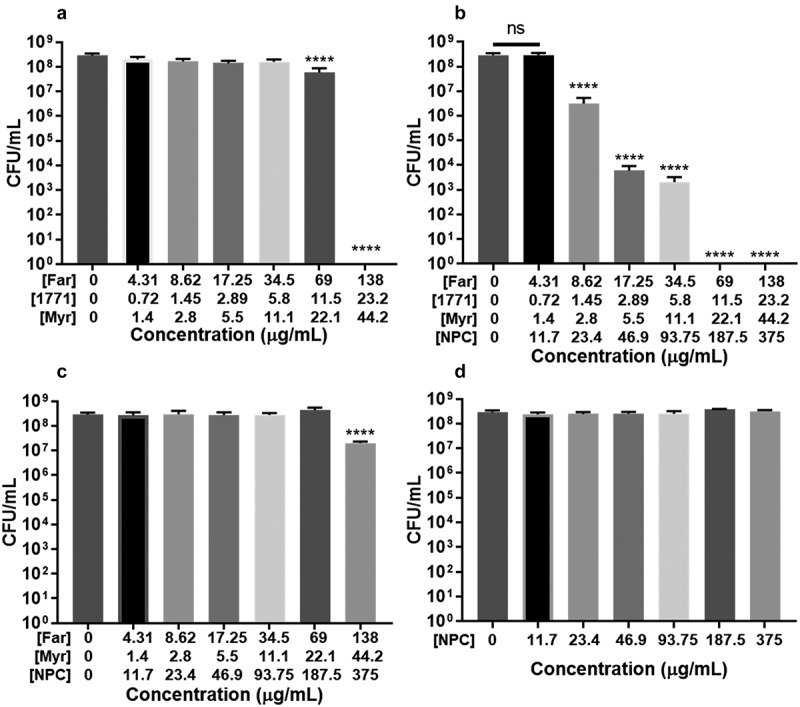
Antimicrobial activity of free and drug loaded-NPC against *S. mutans* UA159 at 10^6^ CFU/mL. Data shown are mean ± standard deviation from three independent experiments using ANOVA with Tukey’s correction for multiple comparisons. (**A**) Association of *tt*-farnesol (Far), myricetin (Myr), and compound 1771 free; ****p < 0.0001 compared to non-treated. (**B**) Association of NPC with *tt-*farnesol, myricetin, and compound 1771; ****p < 0.0001 compared to non-treated and ns is p > 0.9999. (**C**) Association of NPC with *tt-*farnesol, and myricetin; ****p < 0.0001 compared to non-treated. (**D**) NPC alone show no statistical differences between groups

Antimicrobial activity of drug loaded NPC against planktonic *S. mutans* was tested versus free drug and NPC controls. NPC-far-myr-1771 reduced the concentration required to inhibit the bacterium grown, compared to free far-myr-1771 (Figure 7A-B). A lower concentration of the three drugs was only effective when it was associated with NPC. Drug concentrations of 138 µg/mL *tt*-farnesol, 44.2 µg/mL myricetin, and 23.2 µg/mL compound 1771 were required to reduce 4 log of CFU/mL (Figure 7A) compared to 17.25 µg/mL *tt*-farnesol, 11.1 µg/mL myricetin, and 5.5 µg/mL compound 1771 associated to NPC (Figure 7B). Compound 1771 in the formulation had the greatest impact on prevention of bacterial growth, especially in combination with *tt*-farnesol and myricetin (Figure 7C). The nanoparticle alone (unloaded NPC) did not inhibit microbial growth; thereby, all effects were due to drug-loaded NPC (Figure 7D). However, NPC formulations had no impact on *C. albicans* growth (Supporting Information, Figure S2).

*Multidrug delivery via NPC prevented biofilm formation against single- and dual-species biofilms and required compound 1771*

A preventive strategy for caries is to hinder the accumulation of microorganisms on the tooth surfaces; thus, the proposed treatment acts directly on the initial unprotected microbial colonizers. However, it is more challenging to control established biofilms because microorganisms are protected by a matrix rich in exopolysaccharides. Therefore, both paradigms were tested here.

**Figure 8.**
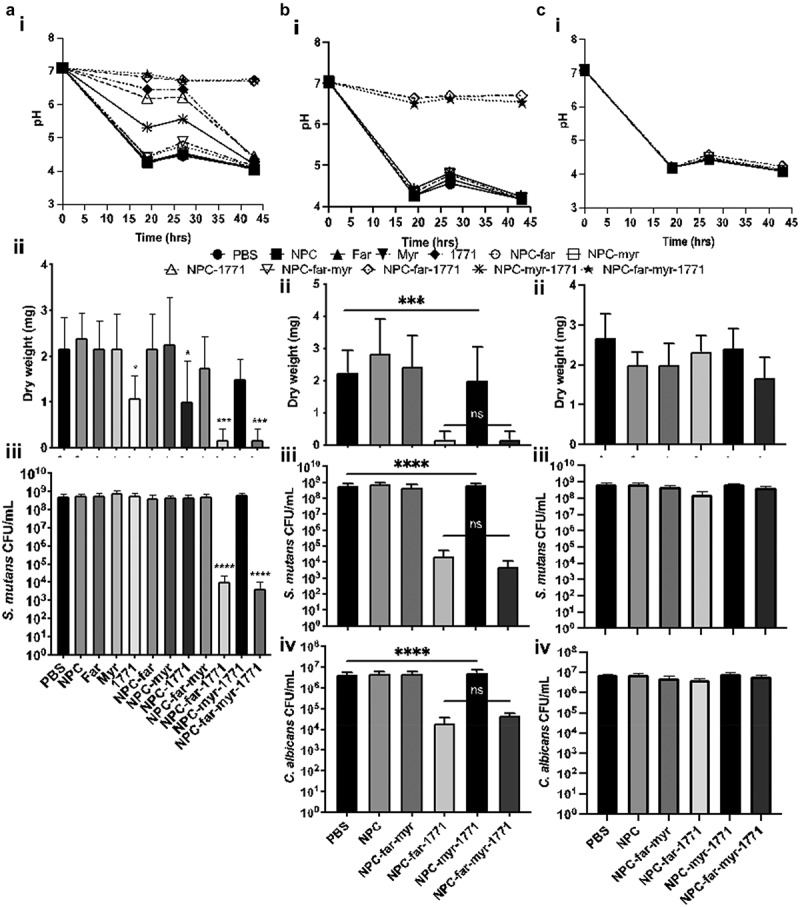
Antibiofilm activity of free or NPC-loaded *tt*-farnesol, myricetin, and compound 1771. (**A-C_i_**) The pH of spent medium over time. (**A-C_ii_**) Biofilm dry weights were obtained for biofilms formed on sHA disks for each treatment. (**A-C_iii_**) *S. mutans* UA159 viable population. (**B-C_iv_**) *C. albicans* SC5314 viable population. (**A**): *S. mutans* single-species biofilm treated preventatively. (**B**): *S. mutans* and *C. albicans* dual-species biofilm treated preventatively. (**C**): *S. mutans* and *C. albicans* dual-species biofilm treated by the control protocol. Data are shown as mean ± standard deviation from three independent experiments in duplicate using ANOVA with Tukey’s correction for multiple comparisons. **** mean p < 0.0001 and ns mean p > 0.0001 or no statistical difference. CFU/mL = colony-forming units per milliliter

In prevention studies, *S. mutans* single-species biofilms were initially tested. Results from these studies showed that all formulations that included 1771 exhibited excellent control of early biofilm growth (depicted by spent medium pH values), but after 27 h, only NPC-far-1771 and NPC-far-myr-1771 maintained pH at or higher than 6.7 (Figure 8Ai). Biofilm dry-weight, or biomass, was reduced for all treatments that included compound 1771 in solution (Figure 8Aii). Less biomass was observed for NPC-far-1771 and NPC-far-myr-1771 treatments; 0.17 mg ± 0.24 mg versus 2.4 mg ± 0.49 mg for unloaded NPC. NPC-far-1771 and NPC-far-myr-1771 treatments also reduced bacteria quantity by four- to fivefold CFU/mL compared with all other treatments (Figure 8Aiii).

NPC formulations were then evaluated using dual-species biofilms comprising *S. mutans* and *C. albicans*. Formulations containing compound 1771 did not uniformly control the pH of the initial biofilm (Figure 8Aii) akin to what was observed in single-species biofilms (Figure 8Ai). However, biofilm pH was controlled using NPC-far-1771 and NPC-far-myr-1771 treatments, with pH maintained at 6.5 or higher (Figure 8Aii). The dry weight and CFU/mL results followed the pH results, showing that NPC-far-1771 and NPC-far-myr-1771 were most effective in preventing dual species biofilm formation (Figure 8Bii), reducing biomass to 0.17 ± 0.24 mg for both versus 2.83 mg ± 0.99 mg for unloaded NPC (Figure 8Biii) and bacterial and fungal growth (four- to fivefold for bacterium and twofold for fungus vs. unloaded NPC – Figure 8Biv).

Established biofilms were also tested to more rigorously characterize NPC-drug formulations. Treatments were universally ineffective against treatment of established biofilms. There was no difference in pH values, dry weight, or CFU/mL of *S. mutans* and *C. albicans* between unloaded NPC and test formulations (Figure 8Aiii-iv).

**Figure 9.**
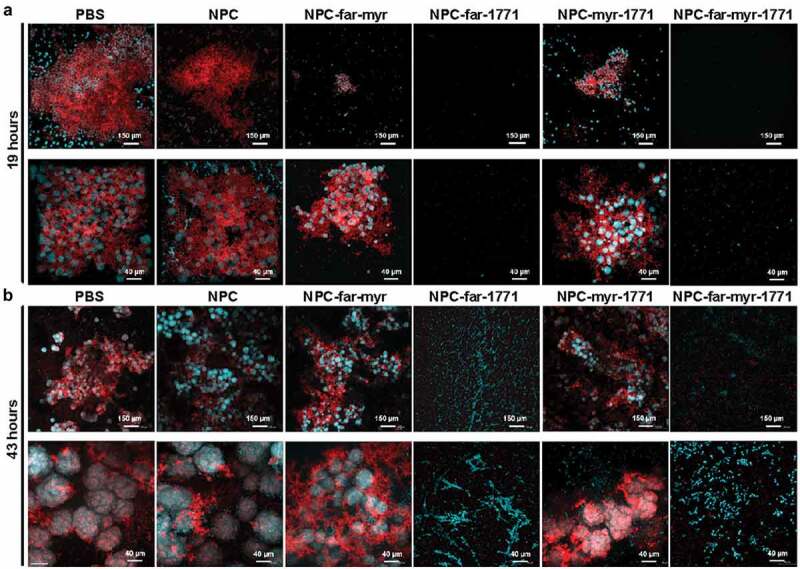
Representative confocal microscopy images of 19 h (**A**) and 43 h (**B**) *S. mutans* and *C. albicans* dual-species biofilms treated using the prevention protocol. The green color represents microorganisms (labeled with SYTO9). The red color represents exopolysaccharides in the extracellular matrix produced by *S. mutans* Gtfs (labeled with Alexa Fluor 647). All images overlay both components using 10 x (top row) and 40 x (bottom row) objective (Andor Dragonfly Spinning Disc Confocal)

**Figure 10.**
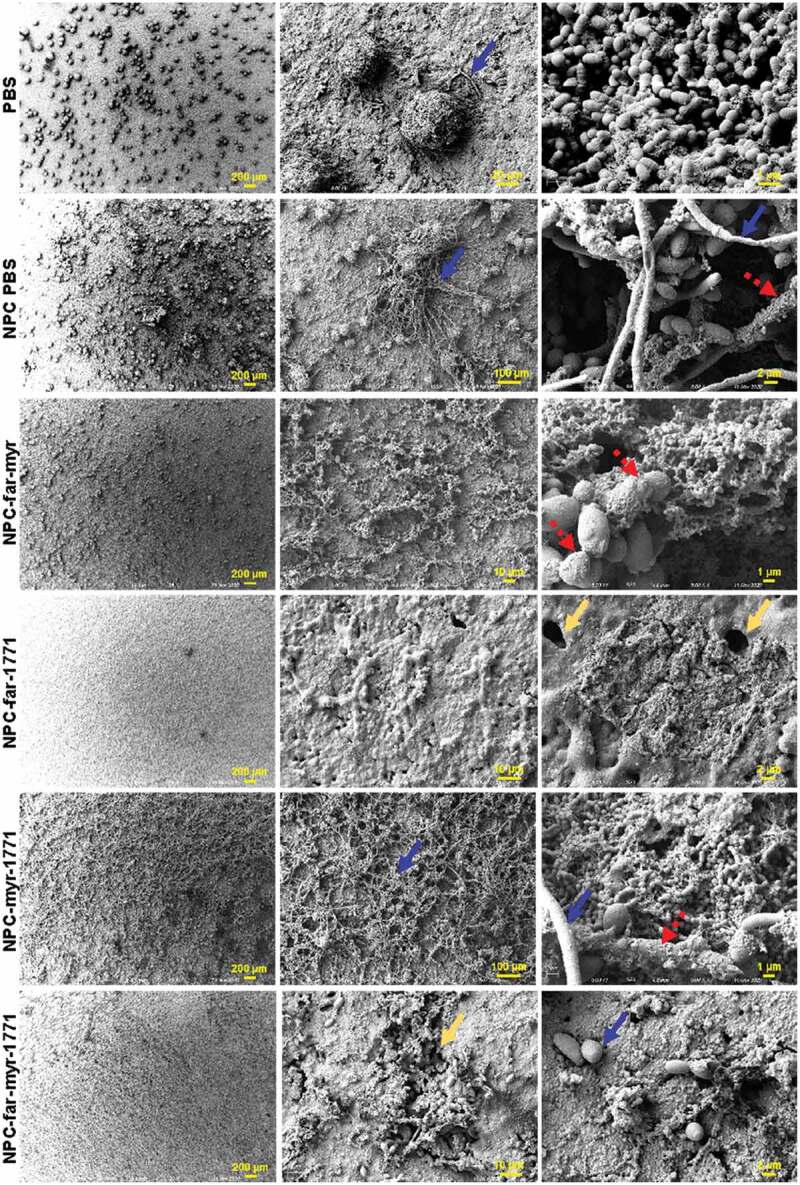
Representative scanning electroscope microscopy images of 43 h *S. mutans* and *C. albicans* dual-species biofilms treated using prevention protocol. Images were captured with different objective lenses to highlight the particularities of each biofilm after treatment and how NPC-far-1771 and NPC-far-myr-1771 reduce biofilm accumulation (Zeiss Auriga field emission and Gatan digital camera system). The yellow arrow represents defects on the hydroxyapatite disc surface; the blue arrow represents the *C. albicans*; the red dashed arrow represents the binding sites on *C. albicans* surface, helping *S. mutans* cells binding and supporting the extracellular matrix

The structure of biofilms treated with the prevention protocol was evaluated at 19 h, representing nascent biofilm, and 43 h, representing mature biofilm, via confocal microscopy (Figure 9) and at 43 h using scanning electroscope microscopy (Figure 10). Biofilms treated with unloaded NPC or PBS (diluent of NPC) exhibited greater and more rapid accumulation of microbes on the HA surface. At 19 h, the biofilm had many cells, and a rich exopolysaccharide matrix that protected these microorganisms, and this community continued growing into mature biofilm [5]. NPC and PBS-treated mature biofilm was similar structurally but with even more robust EPS. NPC-far-myr and NPC-myr-1771 treatments delayed maturation of biofilms, which had fewer microcolonies at 19 h compared to PBS and unloaded NPC. After 43 h, NPC-far-myr and NPC-myr-1771 treated biofilms exhibited similar complexity to PBS controls. Thus, NPC-far-myr and NPC-myr-1771 treatments delayed biofilm formation but did not ultimately hinder colony formation at the latter time point. In contrast, there were only a few isolated microorganisms and no EPS accumulated for NPC-far-1771 and NPC-far-myr-1771 formulations at either time point. Therefore, these two formulations did not just delay but also impeded biofilm development. The amount of biofilm and the difference in biomass were even visible without magnification (Supporting Information Figure S3).

The images from scanning electron microscopy confirmed observations from confocal microscopy (Figure 10). PBS treatments showed spherical microorganisms adhering to each other enmeshed within an exopolysaccharide protective matrix. Interestingly, treatment with NPC alone increased *C. albicans* growth and matrix production. NPC-far-myr treatments had substantial exopolysaccharide production compared with PBS and less apparent *C. albicans* morphological changes compared with NPC and NPC-myr-1771. NPC formulations that included *tt*-farnesol induced *C. albicans* to maintain the yeast phenotype rather than transitioning to hyphae. NPC-myricetin-1771 treated biofilms showed substantial exopolysaccharide production and *C. albicans* hyphae compared to all other treatments tested, highlighting the importance of *tt*-farnesol to control the fungal phenotype. NPC-far-1771 and NPC-far-myr-1771 treatments resulted in unstructured biofilms with only a few remaining microorganisms and minimal exopolysaccharide matrix, similar to previously described quantitative and qualitative data. In fact, most cells were found within HA disk surface defects (Figure 10 – yellow arrow).

## Discussion

Previous studies showed that *tt*-farnesol [35], myricetin [39], and compound 1771 are promising treatment strategies for oral biofilm prevention [41]. The combination of *tt*-farnesol, myricetin, and compound 1771 with and without fluoride was also advantageous for preventing cariogenic biofilms [55]. However, these small-molecule drugs required long exposure times for effectiveness or were only tested in single-species biofilms. Regarding substantivity, reducing the exposure time to a period similar to that of a mouthwash and maintaining or increasing anti-biofilm efficacy are imperative for translation of antibiofilm technology. NPC-mediated drug delivery addressed substantivity, in which drug retention was controlled by cationic surface characteristics and rapid and robust surface binding and thereby enabled a reduction in exposure time (here, 1.5 min compared with 5–10 min) [29–31]. Drug-loaded NPC maintains electrostatic binding to saliva-coated hydroxyapatite, matrix, and microorganism surfaces with no or minimal impact of drug loading within NPC cores [30,31] or coronas [48]. Here, the same diblock copolymer was used to load compound 1771 alone or in combination with *tt*-farnesol and myricetin. Combinatorial formulations maintained high drug loading and electrostatic-binding capabilities. In formulations including NPC-far-myr and NPC-far-myr-1771, the cationic corona, which releases myricetin and/or compound 1771, and pH-sensitive cores, which releases *tt*-farnesol, increased substantivity through binding to the target site (hydroxyapatite surface or exopolysaccharide surface) and sustained release of drug, which effectively abolishes biofilm build-up in prevention studies. However, NPC treatments had no effect on fully established biofilms.

NPC-drug interactions were evaluated using absorbance and fluorescence spectroscopy to estimate drug-binding constants [48]. Similar to the data reported here for 1771, myricetin was found to bind within the NPC coronas based on drug absorbance shifts [48]. However, upon loading compound 1771, binding site competition or mitigation of electrostatic interactions at the NPC corona could reduce the efficiency of myricetin co-loading and binding of the drug-loaded NPC to the salivary pellicle, exopolysaccharides in the matrix, and/or microbial surfaces. Nevertheless, by tuning drug loading, competition was mitigated, resulting in expected color changes from yellow to dark purple (electrostatic interaction between NPC-myricetin) (Figure 2D) and size (Figure 5A-B) as well as positive surface charge (Figure 5C-D). Thus, presence of compound 1771 in the NPC corona did not alter the electrostatic interaction between NPC and myricetin, which contributed to preventing *S. mutans* growth (Figure 7B vs. 6 C).

The observation that treatments maintained pH at neutral levels (7 > pH > 6.5) clarified the underlying mechanism of combinatorial drug efficacy (Figure 8Ai-ii). NPC released 90% of compound 1771 (Figure 6A-B) and myricetin after 6 h independent of pH, followed by ~60% *tt*-farnesol at 6 h and ∼90% at 24 h, as observed previously for co-loading of myricetin and *tt*-farnesol [29]. More rapid release occurred at low pH (critical pH of 4.5–5) when NPC micellar structure was disrupted due to electrostatic repulsion, resulting in rapid drug release within the acidic biofilm niche [30]. In addition, compound 1771 can inhibit lipoteichoic acid synthesis, compromising Gram-positive cell walls [42,43] and disrupting microbial co-aggregation (Figures 9 and 10 – NPC-far-1771 and NPC-far-myr-1771 treatments), which is fundamental for biofilm formation [5]. Reduced biofilm biomass (dry weight) found for NPC-drug treated *S. mutans* single-species biofilm indicated that these two formulations affected the cohesion of bacterial cells and adhesion to the pellicle. Also, bacterial cell counts for single-species biofilms were lower than for the other formulations. However, the magnitude of the reduction compared to biomass was less pronounced because a weakened matrix can favor microbial cells dispersal during biofilm processing via sonication, as observed previously for *gtfB* null *S. mutans*, which exhibited decreased EPS production [56].

Polymicrobial biofilms containing bacteria and fungi such as *S. mutans* and *C. albicans* are more challenging to control [50,51] because of their complexity [55]. However, delivery of NPC-far-1771 and NPC-far-myr-1771 prevented biofilm formation, reduced dry weight, maintained near-neutral pH of spent media (Figure 8B), and eliminated the overall microbial population. Myricetin inhibits F-ATPase activity and genes that encode this proton pump associated with aciduricity; it also affects Gtfs by decreasing gene expression and the exoenzymes activity, thereby reducing glucan formation [29,51]. Thus, treatment with compound 1771 and myricetin in the early stages of biofilm formation impeded microbial co-aggregation and production of glucans to initiate microcolonies. This effect was complemented by *tt*-farnesol, which reduces acid tolerance and several genes responsible for stress tolerance (*sodA, sloA*, and *copY*) and glucan production (e.g. *gtfB*) [35,51]. *tt-*farnesol is a quorum sensing molecule produced by *C. albicans* that promotes the yeast morphology [36], and can also induce fungi apoptosis [57].

There were substantial differences in formulation efficacy. For example, controlling lipoteichoic acids and co-aggregation via compound 1771 delayed biofilm build-up and prevented 3D organization, overall reducing virulence (NPC-far-1771 and NPC-far-myr-1771). However, NPC-myr-1771 had less impact on *C. albicans* compared with NPC-far-1771 formulations. The treatments that did not incorporate *tt*-farnesol showed more *C. albicans* in the hyphae and pseudo-hyphae phenotype, which increase biofilm virulence [10,58]. Fungi protected and supported microcolonies and provided binding sites for *S. mutans* and matrix (as observed in the confocal microscopy and scanning electron microscopy images – Figures 9 and 10). While previous studies have shown that NPC-far and NPC-far-myr treatments controlled *S. mutans* single-species biofilms [29,30,46], the complexity of the dual-species further amplified by shorter exposure times outstrips treatment efficacy [29].

NPC provides highly effective controlled and localized release of drugs to prevent biofilm formation. However, the oral microbiota has several species and the biofilm formed *in vivo* is much more complex than the one tested here. For this reason, myricetin may be critical to include even though NPC-far-1771 exhibited similar efficacy compared to NPC-far-myr-1771 because of its distinct targets, including streptococcal Gtfs that bind to several microbial species and salivary pellicle [8]. Thus, myricetin could aid in the prevention of more complex biofilms in the oral cavity. The control protocol importantly showed that topical treatments had no efficacy on established biofilms; they did not kill microorganisms, just reduced cell colonization of the surface by reducing co-aggregation during the early stages of biofilm development. The topical treatments target early adhesion, matrix formation, and fungal morphological changes. After the biofilm is established on the surface and produces a matrix to protect microbes, it is infinitely more difficult to hinder the maturation process. Notwithstanding, all standard topical treatments, such as fluoride and chlorhexidine have the same challenges [59]. Importantly, previous studies verified that the agents and the nanoparticle carrier were not cytotoxic *in vitro* against the NOK-si cell line [41] or *in vivo* in a rodent model of dental caries [as noted by the absence of oral mucosal damage; 30, 51].

In conclusion, combinatorial formulations were explored to prevent pathogenic biofilm build-up. NPC-far-myr-1771 was successful in preventing both single and dual-species biofilms. NPC-mediated delivery of *tt*-farnesol and compound 1771 is crucial for the prevention regimen. Still, myricetin should not be discarded, as more complex biofilms may demand myricetin anti-Gtf activity to ensure efficacy. The NPC system was vital to increase the drug efficiency by maintaining the drugs for more time in contact with microorganisms.

## Supplementary Material

Supplemental Material
