# Supplementary material for: Nanoparticle carrier co-delivery of complementary antibiofilm drugs abrogates dual species cariogenic biofilm formation in vitro
Source: J Oral Microbiol. 2021 Nov 25;14(1):1997230. doi: 10.1080/20002297.2021.1997230 (PMC8635615; doi:10.1080/20002297.2021.1997230)
Supplement: Supplemental Material [file ZJOM_A_1997230_SM2959.docx]

**Supplemental Figures**

Figure S1. Representative HPLC spectra are showing elution peaks for compound 1771 (∼6.5 min).

**
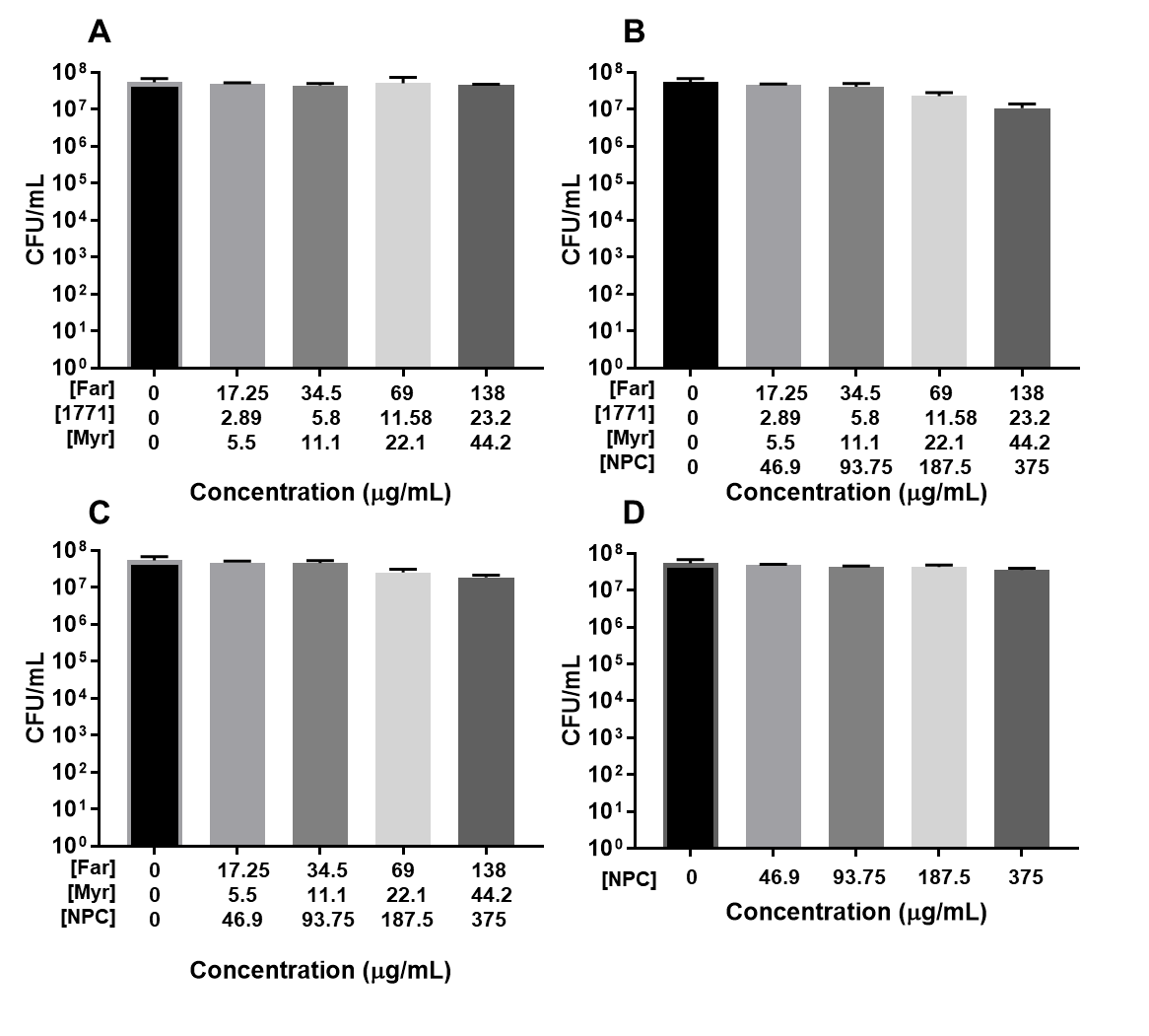
**

**Figure S2.** Antimicrobial activity of drugs associated or not with NPC against *C. albicans* SC5314 at 10^4^ CFU / mL. None of the tested solutions inhibited *C. albicans* growth. (**A**) Association of *tt*-farnesol, myricetin, and compound 1771 free (not loaded into NPC). (**B**) Association of NPC with *tt-*farnesol, myricetin, and compound 1771. (**C**) Association of NPC with *tt-*farnesol and myricetin. (**D**) NPC alone. Data shown are mean ± standard deviation from three independent experiments.


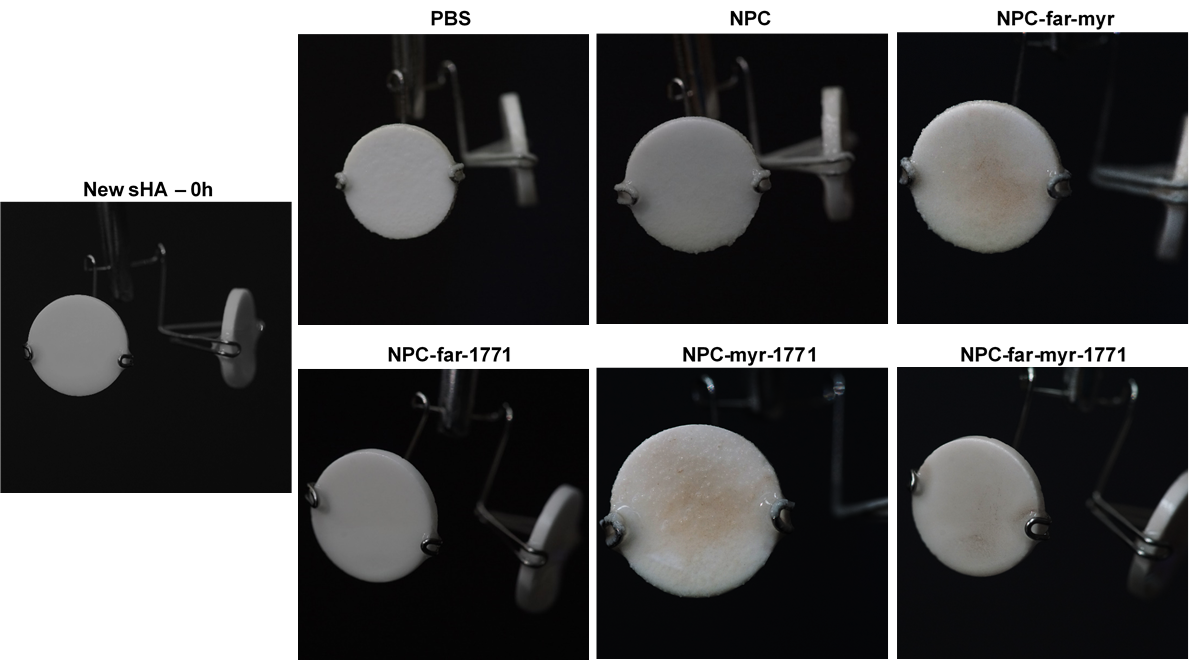


**Figure S3.** Images from 43 h-old HA discs treated using a prevention protocol against *S. mutans* and *C. albicans* dual-species biofilm. The yellowish biofilm is characteristic of the treatment containing myricetin; even for the NPC-far-myr-1771 visually clean, it is possible to see the stain. NPC-far-1771 and NPC-far-myr-1771 showed very similar~~ly~~ to new sHA discs, not just on the disc surface but in the metal clip too. All surfaces are clean compared with other treatments. PBS, NPC, NPC-far-myr, and NPC-myr-1771 are full of biofilm on the disc and on the metal wire. The camera was prepared to take photos at 1/100 s; F/6.3; ISO-1000 - sRGB (Camera Sony - model: ILCA-77M2 – 100mm F/2.8 STF macro lens).
